# Community acquired bacterial meningitis in Cuba: a follow up of a decade

**DOI:** 10.1186/1471-2334-10-130

**Published:** 2010-05-25

**Authors:** Antonio E Pérez, Félix O Dickinson, Misladys Rodríguez

**Affiliations:** 1 Department of Epidemiology, Institute "Pedro Kourí", Autopista Novia del Mediodía Km, 6 1/2 Municipio La Lisa, Ciudad Habana, 17100, Cuba

## Abstract

**Background:**

Community acquired Bacterial Meningitis (BM) remains a serious threat to global health. Cuban surveillance system for BM allowed to characterize the main epidemiological features of this group of diseases, as well as to assess the association of some variables with mortality. Results of the BM surveillance in Cuba are presented in this paper.

**Methods:**

A follow up of BM cases reported to the Institute "Pedro Kourí" by the National Bacterial Meningitis Surveillance System from 1998 to 2007 was completed. Incidence and case-fatality rate (CFR) were calculated. Univariate analysis and logistic regression were used to elucidate associated factors to mortality comparing death versus survival. Relative Risk (RR) or odds ratio and its 95% confidence interval (CI 95%) were estimated, using either a Chi-squared Test or Fisher's Exact Test as appropriate. A Holt-Winters model was used to assess seasonality.

**Results:**

4 798 cases of BM (4.3 per 100 000 population) were reported, with a decreasing trend of the incidence. Highest incidence was observed in infants and elderly. Overall CFR reached 24.1% affecting mostly older adults. *S. pneumoniae *(23.6%), *N. meningitidis*(8.2%) *and H. influenzae*type b (6.0%) were the main causative agents. Males predominate in the incidence. Highest incidence and CFR were mainly clustered in the centre of the island. The univariate analysis did not show association between delayed medical consultation (RR = 1.20; CI = 1.07-1.35) or delayed hospitalization (RR = 0.98; CI = 0.87-1.11) and the fatal outcome. Logistic regression model showed association of categories housewife, pensioned, imprisoned, unemployed, *S. peumoniae *and other bacteria with mortality. Seasonality during September, January and March was observed.

**Conclusions:**

The results of the National Program for Control and Prevention of the Neurological Infectious Syndrome evidenced a reduction of the BM incidence, but not the CFR. Multivariate analysis identified an association of mortality with some societal groups as well as with *S. peumoniae*.

## Background

Despite the advances in antibiotic therapy and vaccines as well as the availability of sophisticated intensive care reached in the past century and the ongoing, community acquired Bacterial Meningitis (BM) remains a serious threat to global health [1,2].

Several issues support its relevance. BM is a leading cause of high morbidity in all ages but young children are at great risk, especially infants. This often fatal disease accounts for an estimated annual 170 000 deaths worldwide and case fatality rates (CFR) remain at 5-10% in industrialized countries, and are even higher in the developing world [1,3-6]. Between 10-20% of survivors develop permanent sequel such as epilepsy, mental retardation or deafness and other neurological disabilities which may contribute to the Global Burden of BM measured as the disability adjusted life years (DALYs) [7].

A great variety of bacteria can cause BM although three species, *Streptococcus pneumoniae*), *Neisseria meningitidis *and * Haemophilus influenzae *type b (Hib) are responsible for most cases of bacterial meningitis occurring beyond the neonatal period, causing more than 80% of cases worldwide [1]. On the other hand a decrease of the susceptibility to conventional and new antibiotics may difficult the treatment [3]. Fortunately there are vaccines against the most common aetiological agents, with high efficacy [1-3], but their use and coverage are unequal depending on public health and economical resources in each country.

Surveillance remains an essential tool to identify the cases, the aetiological agents, and other important epidemiological variables. Routine surveillance systems differ according to the different regions. In the developed world they are mostly based on mandatory notifications of infectious diseases and more recently on laboratory [8-11], while in developing countries are based on case reports and epidemics notifications [12], as well as on hospital sentinel surveillance [13].

First cases of cerebrospinal meningitis in Cuba were reported in 1916. Since 1961 a Mandatory Report of Diseases, including BM, was implemented by Public Health Ministry. Later in 1979 and during an invasive meningococcal disease epidemic, due to serotypes B and C, a special nationwide surveillance known as Direct Information System arose with the interest to achieve a completeness and timeliness in reporting cases [14,15]. In 1983 it was added an automated questionnaire for reporting. Afterwards, in 1998, all causative bacteria were put under surveillance through an updated questionnaire including complementary information about confirmed cases and different of those routinely collected, remaining as the national data source for the National Program for Control and Prevention of the Neurological Infectious Syndrome (NPCPNIS) [16].

The aim of this paper was to characterize the main epidemiological features of BM in Cuba during a ten year period, as well as to assess the association with mortality of some variables reported by surveillance.

## Methods

### Surveillance for Bacterial Meningitis

We included in the study 4798 confirmed cases of BM reported to the Institute "Pedro Kourí" (IPK) by the National Bacterial Meningitis Surveillance System (BMSS) from January 1, 1998 to December 31, 2007, including all causative bacteria and considering the date of the symptoms onset.

The case definition was "a clinical meningeal syndrome, through the identification of the causal agent directly by culture blood, petecchias, cerebrospinal fluid (CSF) analysis or indirectly by polymerase chain reaction, latex test or another rapid diagnostic test. The cases with negative culture or bacteriological test, but the cyto-chemical exam of the CSF suggested bacterial infection, were considered BM of "unknown aetiology" [17]. Reported cases due to *N. meningitidis *included both, meningitis and septicaemia. Standardized case-report form including information on demographic, clinical presentation, symptoms onset, first medical consultation, hospital admittance and decease date, some occupational-related categories (housewife, student, boarding student, pensioned, imprisoned, military, children attending day care centres, children at home, recluse, unemployed, worker) and other epidemiological data, was completed for each case by provincial epidemiologists.

The present study did not require approval from an Ethics Committee. The Cuban Ministry of Public Health is the governmental organization responsible for the collection of infectious disease notifications, hospital discharge records and population or laboratory surveillance. The management of these data for public health purposes does not require a patient's informed consent nor does it require any authorization regarding privacy laws in Cuba.

### Statistical analysis

Cuban population estimates based on the national census for the years 1998 through 2007 were obtained from the Office for National Statistics in Cuba. A tendency to homogenization is among the main demographic characteristics of Cuban population, especially in the last four decades; therefore the age composition of population groups is very similar in all the regions. On the other hand, in Cuban population there are no racial, indigenous, religious or other social groups different to the main population.

Incidence per/100 000 population and case-fatality rate (CFR) per/100 cases were calculated according to the age-group, gender and provinces.

Age group selection in children and adolescents 1-19 year olds was made on the basis of the attendance to main educational levels: 1-5 years olds attend Day Care Centres (DCC), those 6-11 year olds attend Primary Schools, 12-14 year olds go to Secondary Basic Schools and those 15-19 year olds attend mostly Pre-university, other Technical and Professional Schools. Population older than 20 years was distributed by quinquennial age groups.

The time series analysis was done by the best adjusted method (linear trend), choosing the minimum mean square error and minimum interval residuals as appropriate test for trend assumption.

A Holt-Winters multiplicative analysis was used to analyse the seasonality by using the monthly reported cases.

Univariate analysis was used to elucidate associated factors to mortality comparing deaths versus survival. For the assessment of the timeliness of the medical attention and hospitalization we defined delayed medical consultation as "the time elapsed between the symptoms onset and the first medical consultation greater than one day" and delayed hospitalization as "the time elapsed between the first medical consultation and hospitalization greater than one day".

Also it was assessed by univariate analysis the association of fatal outcome with some host factors (male, children attending DCC, children at home, student, boarding student, housewife, pensioned, military, imprisoned, worker, unemployed, *S. pneumoniae, H. influenzae *type b, *N. meningitidis*, other identified bacteria and non-identified agent). Association was estimated through Relative Risk (RR) and its 95% confidence interval (CI 95%). Age was excluded because it was not possible to rise solid hypothesis allowing dichotomising the rank associated to death, but in regression analysis exact age was included.

Additionally, it was carried out a multiple logistic regression analysis to variables with RR>1. The model was fitted including the selected variables and subsequently dropped one by one until only those that were associated (OR>1.6). We used Epi Info 2002 database, NCSS 2000-PASS 2000 statistic software and Excel (version 5.1) for the statistical analysis, and Microsoft Office Word 2003 (11.5604.5606) as the text processor program.

## Results

### Morbidity

During the study period a total of 4798 cases of BM was reported for an annual average of 480 patients and incidence of 4.3/100 000 population. Comparison of the annual incidence in 1998 (5.5/100 000 population) with 2007 (3.1/100 000 population), showed a significant decrease (43.6%, p < 0.01) and therefore, a decreasing trend (y = -0.2957x + 5.8867; Mean Square Error = 0.2767 and -1 to 1 as residuals interval), with an incidence forecast of 2.6 and 2.3 for the next couple years (Data not shown).

All age groups decreased the incidence with the exception of 12-14 and 45-49 years old, which contrarily increased slightly the incidence 5.3% and 26.1% respectively. The highest incidence reductions of the period were observed in older than 84 group (74.0%), 65-74 year olds (65.3%), 1-5 year olds (60.0%) and infants (58.9%) (Table 1).

**Table 1:** Annual incidence of bacterial meningitis, Cuba, 1998-2007

Age	1998	1999	2000	2001	2002	2003	2004	2005	2006	2007
< 1	81.2	66.7	84.1	56.5	40.5	47.8	43.5	51.2	34.5	33.4
1-5	19.0	16.0	11.1	8.8	4.8	6.4	7.4	9.3	5.8	7.6
6-11	5.0	7.9	4.1	3.6	3.4	5.0	2.9	5.7	3.1	4.0
12-14	2.2	5.9	3.7	3.6	4.3	4.2	2.8	2.2	3.3	3.4
15-19	3.7	4.5	4.5	3.0	1.8	1.9	2.7	4.3	3.0	2.5
20-24	2.8	2.5	1.7	3.9	2.2	1.9	2.0	3.3	1.4	1.5
25-29	2.1	2.7	3.0	1.8	1.2	2.2	2.0	3.5	1.5	1.4
30-34	1.8	2.2	2.7	0.9	1.4	1.0	2.2	2.2	1.8	1.4
35-39	2.4	2.1	2.5	1.4	1.9	2.5	1.5	2.8	1.7	2.2
40-44	2.4	2.8	2.8	1.4	2.7	2.7	3.7	2.0	0.8	1.3
45-49	1.7	3.0	2.2	3.4	3.1	3.2	1.8	2.8	2.2	2.3
50-54	3.2	4.2	3.8	3.6	4.0	3.0	3.9	3.6	3.9	2.6
55-59	5.6	5.0	4.6	5.0	3.4	2.8	2.9	4.1	2.3	3.0
60-64	3.3	6.8	6.0	4.7	3.4	3.7	4.3	5.5	2.3	3.0
65-74	7.5	7.7	7.3	6.5	5.0	4.1	4.4	4.8	3.3	2.6
75-84	9.0	10.7	7.2	10.5	7.3	6.6	5.2	5.7	6.4	6.9
> 84	7.7	9.0	13.4	13.1	9.3	3.6	4.2	6.1	9.6	2.0

**Total**	**5.5**	**6.0**	**5.3**	**4.4**	**3.5**	**3.7**	**3.6**	**4.5**	**3.0**	**3.1**

**Figure 1:**
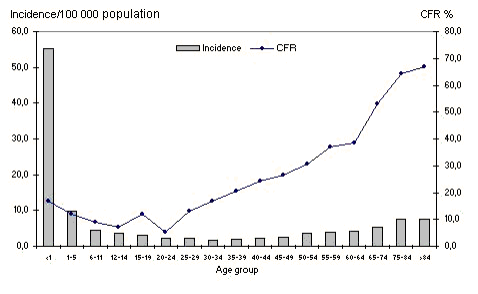
**Average annual incidence and case-fatality rate by age groups**. Cuba. 1998-2007.

With the exception of children under 11 year olds and adults above 74 year olds, all age groups showed an incidence equal or below 6/100 000 population. The highest annual average incidence by age group was observed in infants and children less than 6 years old. The elderly also showed high incidence rates, especially the older than 74 years. The age group with lower rate was 30-34 year olds with 1.7/100 000 population. In the majority of the age groups annual incidence varied considerably along the period (Figure 1).

One thousand four hundred twenty nine cases (39.8%) were younger than 6 years, of them 737 under 1 year old (15.4%) and 692 were aged 1-5 years old (14.4%). These were the most numerous age groups of all. In infants the mean age was 5.3 months, the median 5 months and the mode 2 months (Data not shown).

### Mortality

For the 1998-2007 period a total of 1157 fatal cases were reported and the overall CFR reached 24.1%. Annually variations were observed, with the highest numbers in 2002 (28.5%) and the lowest in 2007 (19.7%) (Table 2).

**Table 2:** Annual case-fatality rate of bacterial meningitis, Cuba, 1998-2007

Age	1998	1999	2000	2001	2002	2003	2004	2005	2006	2007
<1	11.5	11.0	18.3	17.9	17.9	30.8	14.5	15.9	15.8	25.0
1-5	8.6	8.5	13.4	16.9	11.4	13.0	15.4	20.3	7.9	7.7
6-11	7.7	11.1	15.0	5.9	6.3	13.6	12.0	2.0	7.4	5.9
12-14	9.1	6.7	10.5	21.1	0.0	4.5	0.0	0.0	13.3	6.3
15-19	11.5	6.1	14.3	8.3	21.4	6.3	8.7	19.4	11.5	9.5
20-24	4.3	0.0	0.0	7.7	13.3	7.7	0.0	4.2	9.1	9.1
25-29	8.7	31.0	100.0	0.0	45.5	0.0	26.7	7.7	0.0	0.0
30-34	15.0	25.0	1.2	30.0	13.3	36.4	17.4	13.6	5.9	0.0
35-39	13.0	23.8	2.1	18.8	33.3	21.4	18.8	20.0	22.2	13.0
40-44	18.8	31.6	2.0	0.0	23.8	20.8	30.6	20.0	33.3	30.8
45-49	50.0	19.0	4.0	17.4	28.6	33.3	25.0	15.8	31.3	18.8
50-54	25.0	57.7	4.7	30.4	30.8	31.6	36.0	8.7	28.0	5.9
55-59	41.4	37.0	3.0	35.7	36.8	31.3	58.8	25.0	28.6	44.4
60-64	28.6	26.7	42.9	36.4	37.5	38.9	50.0	39.3	41.7	46.7
65-74	63.8	46.9	40.4	48.8	60.6	50.0	54.8	55.9	70.8	42.1
75-84	77.4	63.2	76.9	69.2	63.0	68.0	60.0	50.0	64.0	44.44
> 84	77.8	63.6	76.5	58.8	75.0	100.0	0.0	0.0	71.4	33.3

**Total**	**21.7**	**22.3**	**25.7**	**25.9**	**28.5**	**26.5**	**26.6**	**20.4**	**26.3**	**19.2**


Overall CFR by age groups showed figures over 30% in those above 49 year olds, especially 65-74 (53.0%), 75-84 year olds (64.3%) and the older than 84 years group (67.0%). Among those aged below 15 years the highest CFR were observed mainly in infants (17.0%) (Figure 1).

### Main causative pathogens

Distribution of BM causative agents was as follows: BM of unknown aetiology 2654 (55.3%), *S. pneumoniae *1131 (23.6%), *N. meningitidis *395 (8.2%), Hib 286 (6.0%) and other identified bacteria 332 (6.9%). Three hundred ninety five cases of Invasive Meningococcal Disease were separated in 304 meningitis (77.0%), 85 septicaemia (21.5%) and 6 (1.5%) both, meningitis and septicaemia. Major other identified bacteria were *Staphylococcus ssp., Streptococcus ssp*., *E. coli *and *Klebsiella ssp*. (Data not shown).

### Gender

Gender analysis of the overall and annually male incidence (5.0 per 100 000 population) showed a significant predominance over female (3.6 per 100 000 population). Contrarily, in overall CFR, female (26.4%) predominate slightly over male (22.4%) as also occurred annually, with the exceptions of years 2003 and 2004 when male CFR exceeded female (Data not shown).

### Geographical analysis

**Figure 2:**
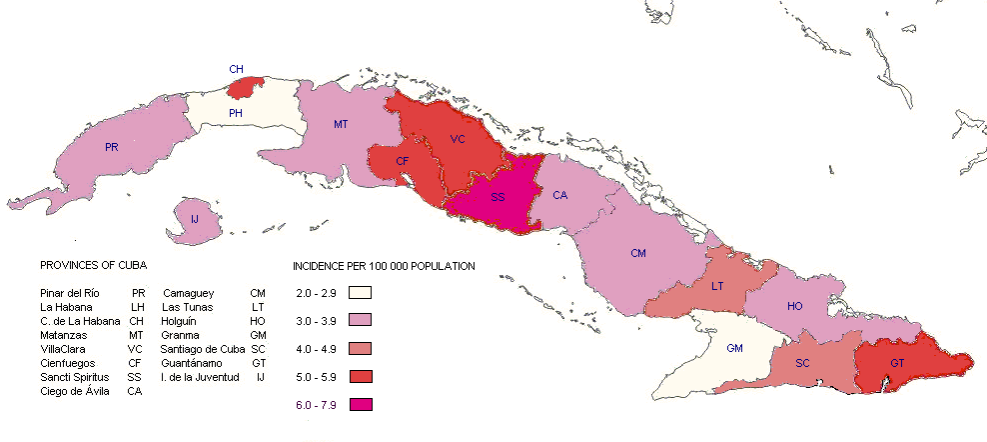
**Bacterial meningitis geographical distribution of the average incidence by provinces. Cuba**. 1998-2007.

Geographical distribution of the average incidence showed that three of the five provinces with higher incidence were clustered in the centre of the island (Villa Clara 5.1 per 100 000 population, Cienfuegos 5.7 per 100 000 population and SS 7.2 per 100 000 population). The Capital City, Ciudad de La Habana (5.0 per 100 000 population) in the western region and Guantánamo in the eastern (5.9 per 100 000 population) also showed high incidence (Figure 2).

Also the higher cumulative CFR was clustered in the central provinces of the island, but towards the eastern side (Cienfuegos 28.8%, Sancti Spiritus 25.4%, Ciego de Avila 32.5% and Camagüey 26.2%). Also Ciudad de La Habana in the western side (27.1%) and Guantánamo (28.9%) in the eastern region showed high CFR (Data not shown).

### Risks

In our study it was possible to obtain the information about the time elapsed between symptoms onset and first medical consultation in 4100 reported cases (85.5%). Of those with available information, a total of 1 139 cases were consulted with delay (27.8%) and 2 961 cases were consulted timely (82.2%). The univariate analysis showed a poor association (RR = 1.20; CI = 1.07-1.35) between delayed medical consultation and death. According to report, 1103 cases were hospitalized with delay (54.1%) and 936 cases (45.9%) were hospitalized timely, but neither an association was found (RR = 0.98; CI = 0.87-1.11) (Table 3).

**Table 3:** Univariate analysis of bacterial meningitis cases for delayed medical consultation and hospital admission with fatal outcome, Cuba, 1998-2007.

		Deceased		RR	Confidence Interval	P-value
		
		Yes	No			
				1.20	1.07-1.35	0.0016
Delayed medical consultation	Yes	318	821			
	No	687	2274			
		**Deceased**		**RR**	**Confidence Interval**	**P-value**
		
		**Yes**	**No**			
				0.98	0.87-1.11	0.7407
**Delayed hospitalization**	**Yes**	264	839			
	**No**	715	221			

Univariate analysis for societal categories only showed association with housewife (OR = 1.98; CI 95% 1.79-2.20), pensioned (OR = 2.65; CI 95% 2.40-2.92), unemployed (OR = 1.68; CI 95% 1.40-2.01), *S. pneumoniae *(OR = 1.62; CI 95% 1.46-1.80), other bacteria (OR = 1.39; CI 95% 1.18-1.64) (Data not shown).

Logistic regression model only showed association (OR>1.6) of the fatal outcome with housewife, pensioned, imprisoned, unemployed, *S. peumoniae *and other bacteria (Table 4).

**Table 4:** Associated factors with the bacterial meningitis mortality.

Variables	Deceased	Survivor	Odds ratio	95% Confidence Interval	P-value
Pensioned	271	222	6.75	5.50-8.28	0.0000
Imprisoned	7	10	4.04	1.51-10.79	0.0052
Housewife	309	434	4.03	3.38-4.81	0.0000
Unemployed	76	114	3.66	2.69-4.99	0.0000
Other identified bacteria	109	223	2.01	1.55 - 2.61	0.0000
*S. pneumoniae*	389	742	1.87	1.59 - 2.20	0.0000

Logistic regression analysis, Cuba 1998-2007

### Monthly distribution

**Figure 3:**
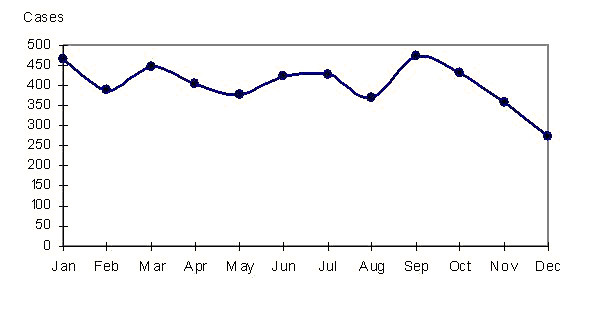
**Bacterial meningitis cumulative monthly mean of cases.** Cuba. 1998-2007

Cumulative monthly mean of cases evidenced three major peak in January (39 cases), March (37 cases) and September (39). The higher number was observed in January and September and the lower number in December (23) (Figure 3). Holt-Winters multiplicative model showed the main increases above monthly mean incidence of the period in September (19%), January (15%) and March (12%), confirming the general seasonality on BM.

## Discussion

Likewise those of developed countries [9-11], BMSS has been useful and proficient providing and synthesizing critical and multidisciplinary information [16,17] for more than a decade, fulfilling all suitable surveillance system attributes [17,18]. Changes implemented in 1998 allowed collecting data which contributed to assess main objectives of both, the National Immunisation Program (NIP) and the NPCPNIS.

However, very few cases of BM might have been missed or misdiagnosed despite the strict control of surveillance and reporting. On the other hand, possible information bias from patients and their relatives was reduced after successive interviews by epidemiologists at hospital and home, as a part of the hot bed control.

Infants and elderly are the most affected age groups by BM and the overall incidence in the study was low and comparable with those of the developed world.

Declining of BM incidence may be the result of the rational use of preventive and control measures nationwide. The impact of vaccination against *N. meningitidis *serotype B and C carried out in 1989 (vaccination campaign targeted to population under 20 years with nearly 95% coverage) and continued in 1991 by the NIP (VA-MENGOC BC^®^, two dose schedule at 3 and 5 months of age), as well as the successful vaccination against Hib carried out in 1999 in children (three dose schedule at 2, 4, 6 months and a booster dose at 18 months) has been reported [19,20]. Therefore, continuous vaccination and high coverage (>95%) achieved in population under 11 years old (Hib) and under 40 years old (*N. meningitidis*) along this period, may explain not only the decreasing trend of the incidence in targeted population, but the decrease in other age groups (herd immunity) [19,20]. At present, *S. pneumoniae *remains the only major causative bacteria of BM in Cuba without specific vaccination control; however the incidence was low and comparable with those achieved by countries that undertook pneumococcal vaccination [21].

Public Health System implemented in Cuba since 1959, is based on nationwide free health service with total access for every citizen to high quality medical assistance, hospitalization, vaccination, social assistance and other health services [19], and undoubtedly also has contributed to BM decreasing trends.

Infants were the most affected group by BM as also occurred in other countries [22,23]. In our study BM was more frequent in this group between two and six months of age, period in which they may have lost the mother protective antibodies and still are not completely protected by vaccines. Other authors report the highest rates of BM among infants between six and twelve months [23,24].

In our series overall and annually male incidence showed a significant predominance over female, coincidently with other author's findings [25,26]. Some sex-specific differences in physical interaction with others and risk behaviour, may explain these findings and will likely require a deeper exploration.

There are a number of stages in the interaction between a sick person and the health care system, at which management decisions may critically affect the speed with which medical treatment is initiated. Delays in receiving appropriate medical care to BM patients more frequently occurs when at home, the signs of disease are not recognised. Once it has been recognised that severity of disease requires medical attention, difficulties may be experienced in consulting with the doctor (not available, low medical attention coverage, lack of transportation, remoteness and others). Once the doctor has seen the patient, the signs of serious illness might not be recognised (lack of familiarity with the condition, inexperience) [27-29] and therefore a delay in hospitalization and specific treatment may occur.

Fatal outcome depends on many factors but care-seeking behaviour in the population, referral practices to hospitals, timely and adequate medical care and specific treatment, the underlying conditions on the patient and the virulence of the strains are the most important [30-34].

NPCPNIS in Cuba recommend the prompt initiation of antibiotic therapy in intensive care unit conditions for all confirmed cases of BM [16]. Regardless of known relationship between increase of mortality and delay in medical attention and hospitalization, that association was not found in our study, showing that timely medical advice and care was received by the majority of patients.

In our study, CFR in infants (17.0%) was coincident to those reported by authors [22,35-37], but high numbers observed, especially among adults [38,39] and older adults [40,41], was probable due to other factors including poor and untimely recognition of symptoms in the elderly.

It was observed association of the fatal outcome with some societal groups (housewife, pensioned, imprisoned, unemployed), as well as the infection caused by *S. peumoniae *and other identified bacteria. These societal groups might be often more vulnerable due to socioeconomic factors and underlying conditions. Also, poor living conditions at home and gathering places as well as the infection by high virulence strains as *Streptococcus *and * Staphylococcus ssp*.might contribute to increase this association. It is important to remark that recently have been reported cases of adrenal haemorrhage as a result of streptococcal infection, evidencing the virulence of this agent [42]. Furthers studies must be undertaken to evaluate more precisely this factors and interactions.

Is interesting how the highest incidence of BM in Cuba is mainly clustered in the central provinces. The Cuban island is long and narrow, and the central region has higher solar radiation and more climate variability than other regions. The combined physical-geographic characteristics and socioeconomic conditions may explain this high incidence [43] and should be more precisely assessed in future studies.

Two well-defined seasons are recognised in Cuba: the rainy or summer (May to October) and the dry or winter (November to April). Since 1990s, climate conditions in winter seasons were projected to be warmer and rainier, and rainy season were projected to be drier and hotter, then this climate conditions are associated with increase of respiratory communicable diseases [43].

Main causative pathogens of BM have each one a peculiar seasonal pattern according to regions of the world. In a study in Lazio, Italy [22], main peaks were observed in January and March, coincident with our results. Seasonal increases of BM observed in our study might be related with seasonal distribution of the main causative pathogens.

## Conclusions

A decade of BM surveillance in Cuba evidenced a reduction in the incidence after using effective preventive and control measures (health education, high coverage vaccination against meningococcus B and C, and Hib, timely hotbed control and others), but still vital efforts are required to decrease CFR, especially in the elderly and other societal groups included in the study.

Future vaccination against *S. pneumoniae *in high risk population will decrease even more the incidence of these lethal infections.

Continuous monitoring of BM and further studies will make possible to assess the need for putting new strategies for the control of these severe groups of diseases in the benefit of the population.

## Competing interests

Authors declares that there is no financial or non-financial (political, personal, religious, ideological, academic, intellectual, commercial or any other) competing interests to declare in relation to the manuscript titled "Community acquired bacterial meningitis: a follow up study of a decade in Cuba".

## Authors' contributions

AEPR: participated in the conception and design of the manuscript as well as in the analysis and the interpretation of data. He performed the statistical analysis, and also was involved in the drafting of the manuscript and its critical revision. He contributed for important intellectual content. In addition, he has the responsibility for the general supervision of the research group. He has given final approval of the version to be published. FODM: participated in the conception and design of the manuscript as well as in the analysis and the interpretation of data. He performed the statistical analysis, and also he was involved in the drafting of the manuscript and its critical revision. In addition he contributed for important intellectual content. He has given final approval of the version to be published. MRO: participated in the acquisition and analysis of data, have been involved in drafting the manuscript and have given final approval of the version to be published.

## Pre-publication history

The pre-publication history for this paper can be accessed here:

http://www.biomedcentral.com/1471-2334/10/130/prepub
